# Prevalence and associated factors of internet addiction among young adults in Bangladesh

**DOI:** 10.1186/s42506-019-0032-7

**Published:** 2020-01-29

**Authors:** Tubayesha Hassan, Mohammad Morshad Alam, Abrar Wahab, Mohammad Delwer Hawlader

**Affiliations:** 1 School of Dentistry, Health Sciences University of Hokkaido, Tobetsu, Ishikari, 061-0233 Japan; 2 Department of Public Health, North South University, Dhaka, 1229 Bangladesh; 3 Health, Nutrition and Population Global Practice, The World Bank, Agargaon, Dhaka, 1207 Bangladesh; 4 Graduex Research Group, Dhaka, 1209 Bangladesh

**Keywords:** Bangladesh, Cell phone, Internet addiction, Social media, Young adult

## Abstract

**Background:**

In the last decades, the use of internet has increased many folds, and internet addiction has become a severe public health issue around the world. This study aimed to determine the prevalence of internet addiction among young adults (19–35 years) in Bangladesh and to identify factors associated with it.

**Methods:**

A total of 454 participants were selected from three administrative divisions of Bangladesh using multistage cluster sampling for this cross-sectional study. A self-reported questionnaire was used to collect data which included Young’s 20 items internet addiction test to assess internet addiction.

**Results:**

The overall prevalence of internet addiction was 27.1%. Addiction rate was 28.6% in the subgroup 19–24 years and 23.5% among 25–35 years old. In both chi-square and logistic regression analyses, internet addiction was significantly associated with living setup, time spent daily on the internet, a detached family relationship, physical activity, and smoking habit (*p* < 0.05). Spending time on social media websites was the most common online activity among the participants.

**Conclusion:**

Our study revealed a relatively high prevalence of internet addiction among younger participants. A detached family relationship and living away from the family were significant determinants along other factors. Therefore, it is important to raise awareness among young generation and their parents towards predictors of internet addiction.

## Introduction

In the present world, the Internet has become an unseparated part of everyday life. The number of internet users, as well as using hours, has grown exponentially among educated people because it is the most appropriate tool for worldwide communication, information source, and a broader source of entertainment [1]. Kimberly Young was the first to introduce the concept of internet addiction disorder (IAD) in 1996 [2], and she recommended including it in the Diagnostic and Statistical Manual of Mental Disorders (DSM), 4th ed [3]. However, the presence of internet addiction (IA) as a mental disorder has not yet been adequately recognized. The Diagnostic and Statistical Manual of Mental Disorders (DSM-5) mentioned that to be considered as a full disorder IAD requires more research. The negative consequences of IAD are visible in daily functioning, family relationship, and social life [4].

Internet addiction is typically described as a state where an individual has lost control of the internet use and keeps using internet excessively to the point where he/she experiences problematic outcomes that negatively affects life [5]. IA is characterized by a maladaptive pattern of internet use leading to clinically significant impairment or distress [6]. Most of the researches identified IA as the use of the internet in a way that creates difficulties in personal life [7]. There has been significant correlation of internet addiction with psychological and interpersonal problems such as inability to relate to other people, loss of control on own behavior, withdrawal from social activities, difficulty to maintain a regular time schedule, and disturbance of sleep and decline in sleep hours [8–10].

In the last decade, there was a nearly sixfold increase in internet usage worldwide, and about 40% of the world is in touch of the internet [11]. In a recent study [12], Xin et al. showed that studies on IA have reported variations in prevalence world-wide. In Europe and the USA, rates ranged from 7.9 to 25.2% among adolescents (2012) while the Middle East and Africa had rates from 17.3 to 23.6%. Studies in Asia have shown a higher variation in prevalence among young people and adolescents, ranging from 8.1 to 50.9%.

In a developing country like Bangladesh, it is necessary to determine the prevalence of internet addiction and identify its associated factors. With the government’s substantial efforts to implement digital technology and revolutionize life on a mass scale, access to the internet is higher than ever. As a result, the number of internet users has increased significantly. The overall figure of internet subscribers has reached 83.141 million at the end of February 2018 which is around 50% of overall population. Among them, 77.495 million are mobile internet subscribers [13]. Although the numbers are justifiable at present, this may turn into a problem for our young-adult population in the future. It is widely known that young adults are the most active internet users worldwide, as they spend approximately 3 h online per day. This overuse is creating various negative impacts which are progressively emerging. The situation has led to significantly increased response against overuse of the internet and its negative consequences all over the world [14].

Most of the studies conducted previously evaluated the prevalence of internet addiction and its predictors in adolescent samples, within the age range of 12 to 18 years. Studies targetting young adults, who are also a high-risk group of internet addiction, are very much limited. Our study aimed to determine the prevalence of internet addiction among the young-adult population and to reveal the associated factors behind internet addiction in Bangladesh.

## Methods

### Study design and tools

This cross-sectional study was conducted in three administrative divisions (Dhaka, Chittagong, and Sylhet) of Bangladesh during the months of January to April of 2018. Young’s 20-item Internet Addiction Scale was used to determine the presence or absence of addiction. A validated Bengali version of the instrument [15] and questionnaire was used to collect information from respondents which were pretested among 5% of the sample population prior to data collection. This widely used instrument has been scientifically analyzed to state an ambiguous psychometric factor structure: salience, lack of control, negligence, time management, etc. Because of the strong correlation among these psychometric factors, it has been considered a robust tool for this study.

To assess behavioral factors, questions were included about the use of internet (length of use, frequency of use, device for use, presence of computer in residence, type of software or application use, type of internet service use, purpose of internet use) and community variables (internet use of friends or colleagues, influence of surrounding people on internet use, influence of surrounding people on internet use). The sociodemographic factors included age, sex, occupation, arrangement of accommodation, area of residence, monthly family income, and personality type. Students were asked to answer questions about family variables (supervision of parents during internet use, problems in family relation, internet use of family members). Cut-off points were marked to categorize the presence of internet addiction. If a respondent got 20–49 points at the scale of 100 points, he was considered an average on-line user. On the other hand, a respondent who scored ≥ 50 points was considered as internet addicted [15, 16].

### Sample size

To estimate the sample size, a single population proportion formula was used. Several previous studies conducted in various locations found the prevalence of internet addiction was around 18% [17, 18]. Considering those reference, the required sample size was *n* = 227 when the allowable error was 5%. Using a design effect 2, the final sample size was 454.

### Sampling and data collection

A multistage clustered sampling was used. From the 3 administrative divisions, 28 districts were targeted, from which three districts (1 from each division) were randomly selected. From each district, two subdistricts were chosen randomly, and finally, households were conveniently selected for data collection. A total of 454 participants were selected (247 males and 207 females). The participants were young adults, internet users, aged between 19 and 35 years. They were categorized according to age, 318 individuals in group A (age 19–24 years) and 136 individuals in group B (age 25–35 years). A Bengali version of questionnaire was filled up by the participants, the first part of which consisted of socio-demographic information and the second part included Young’s 20-item internet addiction test. Participants who did not want to participate were excluded from this study.

### Statistical analysis

Data were checked for completeness and consistency. IBM SPSS version 23 statistical package software was used for data management and analysis. Various descriptive statistics like frequencies and proportions were calculated. Degrees of association between the outcome variable and independent variables were determined by the chi-square test. Variables that showed significant association in chi-square analysis were subjected to multiple logistic regression analyses to explore the strength of association. Results with *p* values of < 0.05 were considered to be statistically significant.

### Ethics approval and consent to participate

Ethical approval for this research protocol was obtained from the Ethics Review Committee of North South University prior to data collection (no. 0013/2018). The aims of the investigation and the nature of the study were fully explained to the participants, who gave informed written consent before participation.

## Results

**Fig. 1:**
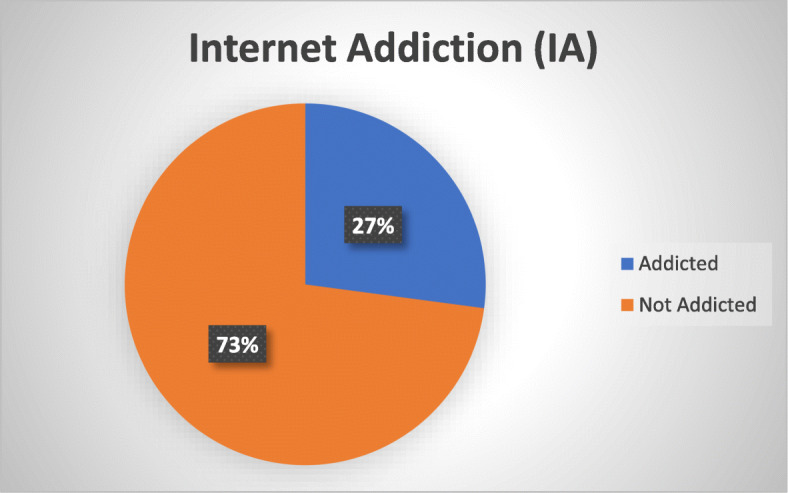
Prevalence of internet addiction among young adults in Bangladesh, 2018

Of the total 454 respondents, 123 (27.1%) were addicted to the internet (Fig. 1).

Most of the participants, 318 (70.0%) were between the age of 19–24 years and 331 (72.9%) of total participants were students. Among the participants, 170 (74.2%) came from the urban area and 34 (7.5%) had a history of family relationship detachment (Table 1).

**Table 1:** Sociodemographic characteristics of young adult internet users in Bangladesh, 2018

Variables with categories	Frequency (No.)	Percent (%)
Age		
19–24 years	318	70.0
25–35 years	136	30.0
Sex		
Female	207	45.6
Male	247	54.4
Occupation		
Housewife	17	3.8
Service	96	21.1
Other	10	2.2
Student	331	72.9
Father’s occupation^#^		
Business	133	29.3
Farmer	38	8.4
Service	180	39.6
Other	38	8.4
Mother’s occupation^#^		
Business	5	1.1
Housewife	377	83.0
Job	28	6.2
Living setup		
Family members	307	67.6
Others (Mess, hostel)	147	32.4
Monthly family income (in taka)		
< 20,000	50	11.0
20,000–40,000	154	33.9
> 40,000	250	55.1
Place of residence		
Rural	44	9.7
Semi-urban	74	16.3
Urban	336	74.0
Having own computer		
No	181	39.9
Yes	273	60.1
Family relationship detachment		
No	420	92.5
Yes	34	7.5
Overuse of internet among family members		
No	321	70.7
Yes	133	29.3
Peers /workplace encouragement		
No	269	59.3
Yes	185	40.7

^#^65 fathers and 44 mothers got retirement or dead

On the other hand, 134 (29.5%) had the habit of smoking (current smoker), most participants (86.8%) were using the internet for more than a year, and 66.5% user used the internet on their mobile phones. Social websites were determined most popular among internet users, and a large number of users (35.2%) liked to stay on the internet for more than 3 h a day (Table 2).

**Table 2:** Activity related characteristics of the young adult internet users of Bangladesh, 2018

Variables with categories	Frequency (No.)	Percent (%)
Recommended amount of physical activity^+^		
No	186	41.0
Yes	268	59.0
Current smoker		
No	320	70.5
Yes	134	29.5
Length of internet use		
< 6 months	34	7.5
6–12 months	26	5.7
> 12 months	394	86.8
Device used to use the internet		
Computer	48	10.6
Mobile phone	302	66.5
Both	104	22.9
Daily internet use duration		
< 1 h	62	13.7
1–< 2 h	117	25.8
2–< 3 h	115	25.3
3 or 3+ h	160	35.2
Most used app/website		
Social	247	54.4
Mailing	89	19.6
Online video	68	15.0
Internet gaming	34	7.5
Other	16	3.5

^+^At least 150 min of moderate aerobic activity or 75 min of vigorous aerobic activity in a week

Addiction rate was 28.62% in the subgroup 19–24 years and 23.53% in the subgroup 25–35 years. Internet addiction was significantly associated with sex (*p* = 0.019), living setup (*p* = 0.012), smoking habit (*p* < 0.01), recommended amount of physical activity (*p* < 0.01), length of internet use (*p* = 0.025), device used to use internet (*p* = 0.027), time spent daily on the internet (*p* = 0.00), family relation detachment (*p* < 0.01), and overuse of internet among family members (*p* = 0.011) (Table 3).

**Table 3:** Association of socio-demographic and activity related characteristics of internet users with internet addiction

Variables with category	Addicted (%)	Not addicted (%)	Chi-square	*P* value
Age			1.25	0.27
19–24 years	91 (28.62)	227 (71.38)		
25–35 years	32 (23.53)	104 (76.47)		
Sex			5.520	0.02*
Male	78 (31.58)	169 (68.42)		
Female	45 (21.74)	162 (78.26)		
Occupation			4.968	0.17
Housewife	1 (5.88)	16 (94.12)		
Job	30 (31.25)	66 (68.75)		
Student	90 (27.19)	241 (72.81)		
Others	2 (20.00)	8 (80.00)		
Father’s occupation			5.004	0.29
Business	45 (33.83)	88 (66.17)		
Farmer	8 (21.05)	30 (78.95)		
Job	46 (25.56)	134 (74.44)		
Others	10 (26.32)	28 (73.68)		
Mother’s occupation			4.028	0.26
Business	3 (60.00)	2 (40.00)		
Housewife	98 (25.99)	279 (74.01)		
Job	10 (35.71)	18 (64.29)		
Living setup			6.359	0.01*
Family members	72 (23.45)	235 (76.55)		
Others (mess, hostel)	51 (34.69)	96 (65.31)		
Monthly family income (Taka)			4.824	0.09
< 20,000	20 (40.00)	30 (60.00)		
20,000–40,000	38 (24.68)	116 (75.32)		
> 40,000	65 (26.00)	185 (74.00)		
Place of residence			0.785	0.68
Rural	14 (31.82)	30 (68.18)		
Semi-urban	18 (24.32)	56 (75.68)		
Urban	91 (27.08)	245 (72.92)		
Smoking (current smoker)			38.201	< 0.01*
No	60 (18.75)	260 (81.25)		
Yes	63 (47.01)	71 (52.99)		
Recommended amount of physical activity			11.233	< 0.01*
No	66 (35.48)	120 (64.52)		
Yes	57 (21.27)	211 (78.73)		
Length of using the internet			7.366	0.03*
< 6 months	6 (17.65)	28 (82.35)		
6–12 month	2 (7.69)	24 (92.31)		
> 1 year	115 (29.19)	279 (70.81)		
Having computer			0.429	0.51
No	46 (25.41)	135 (74.59)		
Yes	77 (28.21)	196 (71.79)		
Device where internet is used			7.223	0.03*
Mobile/tab	35 (22.58)	120 (77.42)		
Computer	50 (25.38)	147 (74.62)		
Both	38 (37.25)	64 (62.75)		
Time spent daily on the internet			67.699	< 0.01*
< 1 h	6 (9.68)	56 (90.32)		
1–< 2 h	12 (10.26)	105 (89.74)		
2–< 3 h	26 (22.61)	89 (77.39)		
3 or 3+ h	79 (49.38)	81 (50.63)		
App/web most commonly used			4.227	0.38
Social media	69 (27.94)	178 (72.06)		
Emailing	19 (21.35)	70 (78.65)		
Online video	20 (29.41)	48 (70.59)		
Gaming	8 (23.53)	26 (76.47)		
Others	7 (43.75)	9 (56.25)		
Family relation detachment			7.418	< 0.01*
No	107 (25.48)	313 (74.52)		
Yes	16 (47.06)	18 (52.94)		
Overuse of internet among family members			6.475	0.01*
No	76 (23.68)	245 (76.32)		
Yes	47 (35.34)	86 (64.66)		
Peers/workplace encouragement			3.641	0.06
No	64 (23.79)	205 (76.21)		
Yes	59 (31.89)	126 (68.11)		

*Statistically significant association at *p* < 0.05

On the other hand, our adjusted analysis indicated that persons who lived with family members were less likely to be addicted (AOR = 0.313, 95% CI 0.17–0.65) in comparison to those who live without family members. The gender of respondents became insignificant in logistic regression model. Participants who spent less time on the internet were also less likely to become addicted to the internet. Moreover, persons who had a good relationship with family members were less prone to be addicted (AOR = 0.098, 95% CI 0.04–0.28) to the internet. Physical activity and smoking habit both were found as potential predictors of internet addiction. Other significant variables in the chi-square analysis such as length of using internet, device used, and overuse of internet among family members did not show statistically significant association with internet addiction in logistic regression model (Table 4).

**Table 4:** Multiple logistic regression analysis of variables associated with internet addiction in Bangladesh

Variables with category	Estimate	Adjusted odds ratio (95% CI)	*P* value
Sex			0.08
Male	0.70	2.031 (0.89–4.61)	0.08
Female	Reference	Reference	Reference
Living setup			< 0.01*
Family members	− 1.182	0.313 (0.17–0.65)	0.002
Others (mess, hostel)	Reference	Reference	Reference
Length of using the internet			0.27
< 6 months	− 0.623	0.622 (0.14–3.21)	0.54
6–12 month	− 1.546	0.236 (0.04–1.56)	0.15
> 1 year	Reference	Reference	Reference
Device where internet is used			0.43
Mobile/tab	− 0.515	0.613 (0.26–1.43)	0.31
Computer	− 0.208	0.824 (0.36–1.92)	0.52
Both	Reference	Reference	Reference
Time spent daily on the internet			< 0.01*
< 1 h	− 3.514	0.033 (0.01–0.13)	0.00
1–< 2 h	− 2.810	0.067 (0.03–0.17)	0.00
2–< 3 h	− 1.622	0.214 (0.11–0.43)	0.00
3 or 3+ h	Reference	Reference	Reference
Family relation detachment			< 0.01*
No	− 2.328	0.098 (0.04–0.28)	0.00
Yes	Reference	Reference	Reference
Overuse of internet among family members			0.51
No	− 0.228	0.796 (0.403–1.571)	0.51
Yes	Reference	Reference	Reference
Recommended amount physical activity			< 0.01*
No	1.12	2.87 (1.65–5.08)	0.00
Yes	Reference	Reference	Reference
Smoking (current smoker)			< 0.01*
No	− 1.41	0.26 (0.13–0.58)	0.001
Yes	Reference	Reference	Reference

*Statistically significant association (*p* < 0.05)

## Discussion

Our research sought to determine the prevalence of internet addiction among young adults of different locations of Bangladesh. Besides, the study also looked for the association between internet addiction and different sociodemographic and internet-usage-related variables.

Our study revealed that the overall prevalence of IA is 27.1%, which is close to a study previously conducted in Bangladesh [19]. The prevalence rates of internet addiction varied across different sociodemographic and internet-use related variables. The current rate (27.1%) was lower than the rates obtained in different Middle East countries like Jordan (40%) [20] and Iran (39.6%) [21] but relatively higher than the studies conducted among British (18.3%) [22] and Taiwanese samples (17.4%) [23]. Besides cultural factors, these differences may be attributed to variations in the diagnostic criteria and assessment questionnaires used for diagnosis. In addition, studies often use highly selective samples of online surveys.

Males were more prone to internet addiction (31.58%) than female (21.74%), which corresponds with previous literatures [12, 24]. It may be because males are generally more passionate regarding knowing the unknown or exploring new inventions or they are usually more attracted to addictive objects such as pornography, cybersex, and online gaming compared with the female [24–26].

Family economic status was revealed as an important determinant of internet addiction in our adjusted model, a good economic condition was found to be inversely associated with internet addiction. A previous Greek study and a study conducted in Bangladesh, 2016, were also in line with these study findings [19, 27]. Increased online engagement is also significantly associated with internet addiction; this online engagement, if not controlled, could appear as problematic, and it is supported by previously conducted studies [28, 29].

On the other hand, people living with family members usually get more time to spend with family members which ultimately gives them support against problematic use of the internet. In our analysis, living setup was found as a strong determinant in our adjusted regression and chi-square analysis.

Internet addiction was also found to be more common among those who had a family relationship detachment. Previously conducted researches also suggest that breakdown of a close relationship is associated with poor mental health by growing gloominess and defeating mentality, which might manifest addictive behaviors as a consequence [30, 31]. Internet addiction was also significantly higher among respondents who had a smoking habit or did not involve in a considerable amount of physical activity.

### Strengths and limitations

The target population of this study was young adults from various regions of Bangladesh whereas previously conducted studies in Bangladesh targeted students of a single region. Also, our study included a considerably high number of sociodemographic variables as well as variables related to internet use behavior and regular activity. Our study also revealed living setup as a significant predictor of internet addiction. This is distinctive in cyberpsychology research in Bangladesh.

There are several limitations as this study is cross-sectional; therefore, no causal relationships were explored. There was a possibility of response-related biases because data were self-reported. Moreover, study participants were conveniently selected for the interview, which may increase potential confounding effects. Despite these limitations, this study had determined the prevalence of IA and investigated the associated factors from a representative sample of several locations in Bangladesh.

## Conclusion

The prevalence of excessive internet use is significant among young adults in Bangladesh, which is conforming with the global trend. There are a number of determinants that have the potential to point out to a much alarming situation. For example, a detached family relationship and smoking habit are strong predictors for internet addiction. Moreover, living setup, time spent daily on the internet, and physical activity level were also significantly associated with internet addiction. As the sample of this study is considerably small, it is insufficient to conclude that internet addiction is high among Bangladeshi young adults. So, further studies using a larger sample size are necessary for proper assessment of the internet addiction situation in Bangladesh. Nonetheless, early awareness is important for the policymakers in order to examine the problem and implement effective measures to prevent it.

## Data Availability

The datasets used in the current study is available from the corresponding author on reasonable request.
